# Soil Water Deficit and Fertilizer Placement Effects on Root Biomass Distribution, Soil Water Extraction, Water Use, Yield, and Yield Components of Soybean [*Glycine max* (L.) Merr.] Grown in 1-m Rooting Columns

**DOI:** 10.3389/fpls.2021.581127

**Published:** 2021-03-15

**Authors:** Michael Gebretsadik Gebre, Hugh James Earl

**Affiliations:** Department of Plant Agriculture, University of Guelph, Guelph, ON, Canada

**Keywords:** soil water deficit, fertilizer placement, drought stress tolerance, soybean seed yield, yield components, volumetric soil water content, rooting profile, rooting columns

## Abstract

Typical small-pot culture systems are not ideal for controlled environment phenotyping for drought tolerance, especially for root-related traits. We grew soybean plants in a greenhouse in 1-m rooting columns filled with amended field soil to test the effects of drought stress on water use, root growth, shoot growth, and yield components. There were three watering treatments, beginning at first flower: watered daily to 100% of the maximum soil water holding capacity (control), 75% (mild drought stress), or 50% (drought stress). We also tested whether applying fertilizer throughout the 1-m soil depth instead of only in the top 30 cm would modify root distribution by depth in the soil profile and thereby affect responses to drought stress. Distributing the fertilizer over the entire 1-m soil depth altered the root biomass distribution and volumetric soil water content profile at first flower, but these effects did not persist to maturity and thus did not enhance drought tolerance. Compared to the control (100%) watering treatment, the 50% watering treatment significantly reduced seed yield by 40%, pod number by 42%, seeds per pod by 3%, shoot dry matter by 48%, root dry matter by 53%, and water use by 52%. Effects of the 75% watering treatment were intermittent between the 50 and 100%. The 50% treatment significantly increased root-to-shoot dry matter ratio by 23%, harvest index by 17%, and water-use efficiency by 7%. Seed size was not affected by either fertilizer or watering treatments. More than 65% of the total root dry matter was distributed in the upper 20 cm of the profile in all watering treatments. However, the two drought stress treatments, especially the mild drought stress, had a greater proportion of root dry matter located in the deeper soil layers. The overall coefficient of variation for seed yield was low at 5.3%, suggesting good repeatability of the treatments. Drought stress imposed in this culture system affected yield components similarly to what is observed in the field, with pod number being the component most strongly affected. This system should be useful for identifying variation among soybean lines for a wide variety of traits related to drought tolerance.

## Introduction

Soybean [*Glycine max* (L.) Merr.] is the number one field crop grown in Ontario, Canada, with a cultivated area of more than 1.3 million ha and a value of over $1.9 billion in 2018 (OMAFRA, 2020). Soybean is grown mostly under rainfed conditions, so soil water limitations occurring during critical stages of crop development significantly reduce Ontario’s soybean yield potential (Hufstetler et al., 2007; Visser, 2014). Recent research results show that soil water deficits constitute a significant limitation to Ontario’s soybean yield in most growing seasons. Demonstrated losses in field experiments ranged from 8 to 24% and supplemental irrigation during the reproductive stage was found to enhance soybean yield by 10–25%, mostly from an increase in number of pods (Earl, 2012; Visser, 2014). Even in unusually wet years, soybean yields in Ontario are reduced by transient soil water deficits, and in drier years, yield losses may exceed 25% (H. J. Earl, unpublished data). Numerous controlled environment and field studies have also reported yield reductions ranging from 24 to 80% in soybean subjected to different levels of drought stress (e.g., Frederick et al., 2001; Sadeghipour and Abbasi, 2012; He et al., 2016, 2017; Wei et al., 2018; Giordani et al., 2019; Gebre, 2020).

In Ontario, such soil water deficits tend to occur during July and August which coincides with the pod-setting and seed-filling soybean developmental stages when the crop is actively growing and daily crop water use exceeds concurrent precipitation. Yield-limiting water deficits in soybean often occur with no obvious outward signs of stress such as leaf wilting, but decrease whole-plant water use (WU) (Earl, 2002; King et al., 2009; He et al., 2016, 2017, 2019; Gebre, 2020; Gebre and Earl, 2020), whole-plant dry matter (DM) (Earl, 2002; He et al., 2016, 2019; Wei et al., 2018; Gebre, 2020; Gebre and Earl, 2020), and also result in fewer pods per plant (Shaw and Laing, 1966; Momen et al., 1979; He et al., 2016, 2017, 2019; Gebre, 2020), reduced seed size (Shaw and Laing, 1966; Momen et al., 1979; Brevedan and Egli, 2003; He et al., 2016, 2017, 2019) and hastened crop maturity, which shortens the seed-filling duration (Westgate and Peterson, 1993; Brevedan and Egli, 2003) and ultimately reduces seed yield (Shaw and Laing, 1966; Momen et al., 1979; De Souza et al., 1997; He et al., 2016, 2017, 2019; Gebre, 2020). King et al. (2009) showed that four soybean genotypes began to decrease their transpiration (water use) rates at soil water contents of 40–43% of maximum transpirable soil water remaining. However, no signs of wilting appeared until that dropped to 23–26% for three of the genotypes, and 18% for the fourth which was a slow-wilting genotype.

Drought stress may limit soybean’s yield through a reduction of any of its yield components (pod number, seeds per pod, and single-seed size). In soybean, yield reduction due to drought stress can occur at any stage of the crop although the magnitude of the yield reduction depends on the stage of development, the timing of the stress, and the severity of the stress. For example, water deficits imposed during the vegetative stage may not have a significant effect on final seed yield, provided that >95% light interception is achieved by the R1 developmental stage (Momen et al., 1979; Van Roekel et al., 2015). However, drought stress during the vegetative stage still causes reductions in internode length, height, stem diameter, and leaf area expansion, which results in a low total (root and shoot) crop dry matter (Bunce, 1978; Hoogenboom et al., 1987a,b; Desclaux et al., 2000; Brevedan and Egli, 2003; Wei et al., 2018; He et al., 2019). The reproductive stages (flowering, pod setting, and seed filling) are much more sensitive to drought stress (Sionit and Kramer, 1977; Morrison et al., 2006; Wei et al., 2018). During these stages, drought stress can significantly decrease the number of flowers (via flower abortion), pods per plant (via pod abortion, commonly the youngest pods), and the number of seeds per pod (Shaw and Laing, 1966; Westgate and Peterson, 1993). The total number of flowers in some varieties may be reduced by up to 50% under drought conditions, reducing the number of pods per plant (He et al., 2019).

When the number of pods is reduced by drought stress, the remaining seeds are often well filled resulting in a heavier (larger) individual seed weight at maturity (Desclaux et al., 2000). When drought stress occurs during the seed-filling stage (R5 to physiological maturity) the number of seeds per pod and individual seed weight is decreased (Desclaux et al., 2000; Brevedan and Egli, 2003; Wei et al., 2018), resulting in flat and empty (non-viable) pods on the upper nodes. Compared to individual seed weight (size), seed number (the product of pods per plant and the average number of seeds per pod) has a greater influence on seed yield, largely via variation in pod number (Egli, 1998; Egli and Bruening, 2006; Robinson et al., 2009). Thus, of the three soybean yield components, the most important determinant of soybean yield seems to be pod number, and this yield component is determined during a period that begins around flowering (R1 or R2) and extends through pod set (R3) and possibly to the beginning of the seed-filling period (R5). These phases are often regarded as the critical period for yield determination (Egli, 1998). Moreover, the number of pods per plant seems to be the yield component that is most strongly affected by drought stress, which ultimately reduces the final seed yield depending on the duration and intensity of the stress period (Shaw and Laing, 1966; Doss et al., 1974). By contrast, the number of seeds per pod and seed size appear to be more stable and genetically controlled yield components (Shaw and Laing, 1966; Van Roekel and Purcell, 2016) which are relatively insensitive to drought stress conditions (Momen et al., 1979).

Most controlled-environment phenotyping studies of soybean germplasm for traits related to drought tolerance are carried out in artificial media in small pots (less than 30 cm tall), where roots easily explore the entire pot volume very rapidly so that rooting traits such as rate of root elongation or final rooting depth have almost no effect on the plant’s ability to access soil water (e.g., Earl, 2002, 2003; Passioura, 2006; Hufstetler et al., 2007; Walden-Coleman et al., 2013). In addition, the root dry matter (DM) distribution and volumetric soil water content (VSWC) profiles in the field vary strongly by depth, whereas in small pots VSWC and rooting profiles are relatively uniform from top to bottom. Drought stress experiments conducted in frequently watered small pots, therefore, may not present soil water and rooting profile variation by depth similar to what is encountered in a field environment. To alleviate those limitations associated with small pots and permit meaningful controlled environment studies of soybean responses to drought stress, and also to capture root DM distribution and VSWC profiles by depth, we developed and characterized a 1-m rooting profile method (Gebre and Earl, 2020). It utilizes amended field soil and better emulates field soil water and rooting profile conditions as they occur in a typical mid-textured agricultural soil.

Similar phenotyping methods (1 m long 20 cm diameter PVC tubes) for drought tolerance have also been reported by others (e.g., Vadez et al., 2008; Ratnakumar et al., 2009) in legumes other than soybean such as in groundnut (*Arachis hypogaea* L.), chickpea (*Cicer arietinum* L.), and pigeonpea (*Cajanus cajan* L.). Specifically, Ratnakumar et al. (2009) used the system mainly to assess groundnut genotypes for their transpiration efficiency, using a large lysimetric system and fully automated rainout shelters developed at the International Crops Research Institute for the Semi-Arid Tropics (ICRISAT).

Extensive literature is available describing roots as potential targets for modification to improve yield and resilience under drought stress (e.g., Earl, 2003; Vadez et al., 2008; Ratnakumar et al., 2009; Ehdaie et al., 2012; Uga et al., 2013; Atkinson et al., 2014; Bucksch et al., 2014; Guo et al., 2016; Fried et al., 2018, 2019), although very few practical achievements have been documented in root-based breeding (Vadez et al., 2008; Ratnakumar et al., 2009; Lynch, 2018, 2019; Schneider and Lynch, 2020). Despite the assumed importance of rooting traits in drought stress responses, effective phenotyping methods addressing root function (as opposed to merely measuring root system morphology) are not widely employed.

Furthermore, many controlled-environment drought stress studies are confined to the vegetative stage (e.g., aboveground DM responses to stress) and so do not provide the opportunity to investigate drought stress effects on yield and yield components (e.g., Hoogenboom et al., 1987a, b; Earl, 2002, 2003; Hufstetler et al., 2007; Fish and Earl, 2009; Walden-Coleman et al., 2013). Growing plants from seed to maturity under controlled environment conditions may provide additional insight into the mechanistic basis of physiological processes affecting yield and yield components. Thus, in the present work there was a desire to characterize how the controlled environment drought stress simulation method affected plant growth and productivity all the way to maturity. A single Ontario adapted elite commercial soybean variety *OAC Bayfield* was used to see how it responded to the different soil water treatments in terms of the growth, yield and yield components, rooting and VSWC profiles by depth. In this system, we were looking for a reproductive phase drought stress protocol that had a realistic and large effect on yield, and where the primary yield component that would be affected was pod number, as is typically the case in the field (Board and Maricherla, 2008; Earl, 2012; Visser, 2014).

Additionally, as another test of the system’s ability to measure belowground (rooting) plant responses to abiotic factors, we introduced another treatment (fertilizer placement) that might affect both root dry matter and VSWC profiles by depth. Fertilizer placement (nutrient availability) may affect the rooting depth, rooting patterns, and soil water extraction in such a manner that crop response to soil water deficits is altered. Hansel et al. (2017) found that fertilizer (phosphorus and potassium; P and K) application rates and placement methods significantly affected soybean yield and drought tolerance, where treatments that promoted deeper plant root systems displayed reduced yield penalties under drought stress conditions mainly due to enhanced water and nutrient acquisition. He et al. (2019) also reported that P application improved soybean yield under different water availabilities by increasing water use during the reproductive stage.

The concentration of P primarily in upper soil layers (as can occur under no-till production systems) when applied as mineral fertilizers may encourage root proliferation near the soil surface, with possible deleterious effects on crop response to soil water deficits. Rooting zones exposed to high P concentrations may cause a localized increase in the initiation and subsequent extension of the primary and secondary roots, compared to those rooting zones experiencing low P concentration (Hansel et al., 2017). Phosphates easily precipitate with soil cations and so effectively have very low solubility, making P highly immobile in soils. Accessing fertilizer P in the soil requires that roots extensively explore the soil volume where the fertilizer has been placed (Grant et al., 2001). Roots of most crops are observed to proliferate in proximity to P fertilizer, so long as the bulk soil is otherwise P-deficient (Hallmark and Barber, 1984). This means that the placement of P fertilizer can affect the distribution of roots in the soil profile (Bonser et al., 1996; Miller et al., 2003; Lynch and Ho, 2005), including in soybean (e.g., Borkert and Barber, 1985; Hansel et al., 2017). If fertilizers are present primarily in upper soil layers, this may discourage deep rooting. Besides, P and K near the soil surface may become unavailable during extended periods of limited precipitation, since the upper soil layers always dry more quickly under those conditions, and P and K uptake cannot occur in the absence of sufficient soil water (Singh et al., 2005). This controlled environment system using 1-m rooting columns presents an opportunity to test those ideas in a scenario where it is easy to measure VSWC and rooting profiles by depth.

We hypothesized that (1) drought stress treatments imposed in this culture system would significantly reduce whole-plant water use (WU), whole-plant dry matter (DM) accumulation, soybean yield, and yield components (pod number, seeds per pod, and single-seed size); (2) considering yield components, pod number would be the main yield component driving the final yield and thus would be most strongly affected by the drought stress treatments, as is typically observed under field conditions; effects on seeds per pod and single-seed size would be very minor; (3) distributing the fertilizer over the whole soil profile would alter the root DM distribution, and that would also affect soil water extraction at different depths of the soil profile, plant growth, seed yield, and yield components. That is, placing the fertilizer throughout the profile would result in more roots at depth which would, in turn, give the plants the ability to extract soil water from the lower depth and therefore be more drought tolerant.

The specific objectives of the present study were to (1) develop and characterize a drought stress simulation method for controlled environment phenotyping of soybean germplasm that has a more realistic belowground environment (1-m rooting columns) than can be achieved in small pot experiments; (2) evaluate the effects of different drought stress protocols on VSWC and rooting profiles by depth, whole-plant water use, whole-plant DM accumulation, soybean yield components (pod number, seeds per pod, and single-seed size) and final yield; and (3) test the effects of fertilizer placement on rooting and VSWC profiles by depth under control and drought stress conditions, in 1-m rooting columns.

## Materials and Methods

### Plant Material and Culture System

Plants were grown in the Crop Science Building’s greenhouse at the University of Guelph (44.5314° N, -80.2244° W), Guelph, ON, Canada, in 2016 using a culture system developed and described in detail by Gebre and Earl (2020). Briefly, a single Ontario-adapted elite commercial soybean variety *OAC Bayfield* was sown on July 15, four seeds per tube at 3 cm depth, and then thinned after emergence to one per tube. *OAC Bayfield* is a commercial soybean variety released to the public in 1993 through SeCan (Kanata, ON, Canada)^1^, and has been successfully grown for over 20 years with excellent yield potential, broad adaptability in Ontario, adequate lodging resistance, and above-average oil content. In controlled-environment studies, it had the highest water use efficiency among seven soybean genotypes and the lowest dark-adapted leaf conductance, g_dark_ (mmol m^–2^ s^–1^), among 63 soybean genotypes tested (Walden-Coleman et al., 2013); both traits that could be related to drought tolerance.

The plants were grown in 1 m long 10 cm diameter PVC rooting columns (tubes) lined with polyethylene liners, and PVC end caps at the bottom with a drainage hole. The soil mixture was a blend of six parts by volume field soil, two parts granitic sand (B-sand; Hutcheson Sand and Gravel Ltd., Huntsville, ON, Canada), and one part peat-based potting mix (PGX; Premier Tech, Brantford, ON, Canada). The field soil used, classified as a “*London loam”* (Gray-brown Podzolic loam till), was collected from the topsoil (upper 15 cm) at the Elora Research Station (Elora, ON, Canada; 44.6837° N, -80.4305° W) that had a prior history of soybean production. It was a silty loam (silt = 50%, sand = 31%, clay = 19%, mineral components by mass) texture and contained 4.2% organic matter, 23.5 ppm P, 61.5 ppm K, 280 ppm Mg, 2,375 ppm Ca, 15 ppm Na, with 14.4 meq 100 g^–1^ CEC, and a pH of 7.4 according to a soil test performed by A&L Laboratories Inc., London, ON, Canada.

The soil mixture loaded into each tube contained a commercial 20-20-20 N-P-K plus micronutrients fertilizer (Master Plant Products Inc., Brampton, ON, Canada) at the rate of 0.8 g tube^–1^. Variation in fertilizer distribution was created by loading the fertilizer (mixed with the soil mixture) just in the top of the profile (0–30 cm; “top loading”) to mimic typical fertilizer distribution in the field, or throughout the soil profile (0–100 cm; “full loading”). In the top loading treatment, the bottom 70 cm of the tube was first loaded with the soil mixture that had no fertilizer added to it; instead, 70 mL of water was sprayed and mixed with the soil mixture. Then, the 0.8 g tube^–1^ fertilizer was dissolved in 30 mL of water and thoroughly mixed with the soil mixture before it was loaded to the top 30 cm of the tube. The fertilizer in the full loading treatment was dissolved in 100 mL water, thoroughly mixed with the soil mixture, and then loaded to the whole tube. During the process of potting, the tubes were filled in a systematic fashion of loading and packing until the soil reached approximately 1 cm below the top part of the tube. The total weight of each tube with its soil was then recorded.

**FIGURE 1:**
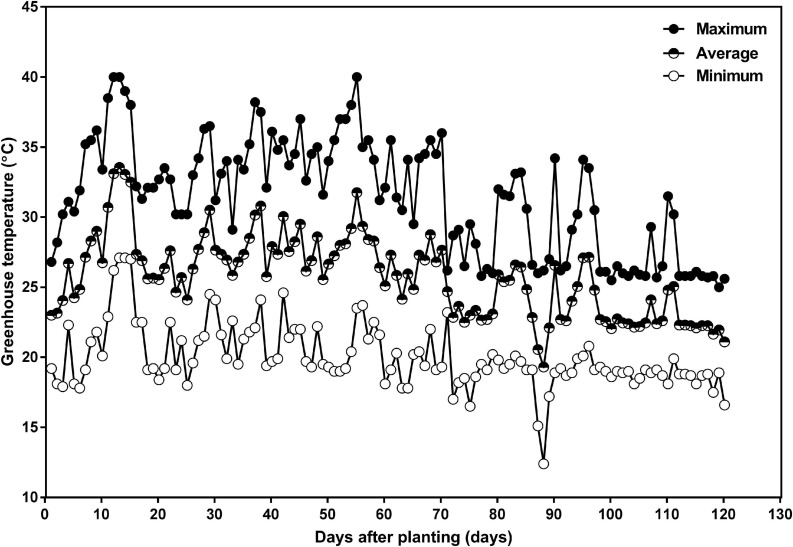
Daily minimum (open circle), average (half-closed circle), and maximum (closed circle) greenhouse air temperature as a function of days after planting in the 2016 summer and fall season, in the greenhouse attached to the Crop Science Building at the University of Guelph. The planting date was July 15 and the harvest date was November 12.

Greenhouse target temperatures were set at 25°C during the day and 20°C during the night with an average relative humidity of 80%. The actual greenhouse daily minimum, maximum, and average temperatures are given in Figure 1. Natural sunlight was supplemented with overhead high-pressure sodium and metal halide lamps to provide a supplementary 400 μmol m^–2^ s^–1^ photosynthetic photon flux density at the top of the canopy during the photoperiod, and to provide daylength extension to achieve 16 h of light and 8 h of dark.

### Determining Soil Water Holding Capacity

To determine the soil water content and mass of dry soil in each tube, soil samples were taken during the potting process and dried in a forced-air drier at 80°C until a constant weight was attained. All the tubes in all treatments were then watered until they started dripping water from the drainage holes. After 24 h, they were watered again to free drainage to ensure that the soil was completely saturated. Elastic bands were used to close the plastic liners at the tops of the tubes to prevent surface evaporation. The tubes were allowed to drain until a constant weight was achieved (tube weight at maximum soil water holding capacity; SWHC). The weights of the dry soil and the tubes were then subtracted from this weight to determine the soil water content at maximum SWHC. Then, the target weight for each tube was calculated as the tube + soil dry weight, plus the water weight at maximum SWHC measured for that tube multiplied by the target fraction of the maximum SWHC (either 100, 75, or 50%, depending on the watering treatment).

### Experimental Design, Treatments, and Measurements

The experiment was arranged as a randomized complete block design, with two fertilizer placement treatments (top loading or full loading) and three watering treatments (watered daily to 100, 75, or 50% of the maximum SWHC), replicated five times (2 × 3 × 5 = 30 tubes). To test the effect of the fertilizer placement on root DM and VSWC distribution, two tubes per replication (2 × 5 = 10 tubes) were also included in the experimental design for a destructive harvest measurement made at the R1 (beginning flowering) developmental stage (32 days after planting; DAP), prior to the initiation of the drought stress treatments (developmental staging as per Fehr et al., 1971; Purcell et al., 2014). These 10 tubes were integrated into the complete randomization in each block; however, they represented a separate experiment and analysis. Once these 10 tubes were harvested, all of the remaining tubes were moved to fill in the gaps created. The 40 experimental units (tubes) were placed on a custom-designed wooden stand, arranged in two rows of 20 tubes. Each replicate consisted of 2 rows × 4 tubes, of which two tubes each of the five replicates were subjected to a destructive harvesting. Four tubes (two at each end) were used to grow border plants to minimize border effects.

All tubes were weighed and watered daily to their maximum SWHC until R1. At this point, watering treatments were imposed and lasted through the R8 (full maturity) developmental stage. During this period (R1 to R8 stages), tubes were returned to either 100% (control), 75% (mild drought stress), or 50% (drought stress) of the maximum SWHC by daily weighing and watering. The whole-plant water use (WU) from planting to harvest was calculated as WU (g plant^–1^) = [total amount of water added to each tube from planting to harvest + (starting weight – end weight of each tube at harvest) + whole-plant fresh biomass at harvest]. The daily cumulative WU (g plant^–1^) was also calculated for each tube on each day as = [(starting weight – current weight) + total amount of water added to the tube up until that point].

Time-domain reflectometry (TDR; Field Scout^TM^ TDR 100 Soil Moisture Meter, Spectrum Technologies, Inc., Aurora, IL, United States) millisecond readings were recorded once a week for the duration of the study, beginning right at the planting date and ending at the R7 (physiological maturity) stage. Before every set of TDR measurements, the TDR meter calibration procedure was performed. The TDR measurements were performed at five equally spaced points (at 10, 30, 50, 70, and 90 cm below soil surface) via the pre-drilled TDR access holes in the sides of the tubes. The TDR measurements were always made just before daily watering (i.e., 24 h after the previous watering). The volumetric soil water content (VSWC; %) was calculated from the TDR millisecond readings using a calibration curve developed for the growth medium.

### Harvest and Postharvest Procedures

About 5–10 days after full maturity (120 DAP; at R8 developmental stage, when 95% of the pods were brown), all plants were cut at soil level and total aboveground plant fresh biomass (stems, branches, pods, and seeds) was recorded. Immediately after harvest, the total number of filled pods (pods with seeds) per plant (pod number; PN) were counted. All these aboveground samples were then placed in labeled paper bags and oven-dried in a forced air drier at 80°C until a constant weight was attained. Then, the total aboveground (shoot) dry matter (SDM) per plant was determined, pods were threshed by hand, seed yield (SY, the weight of seeds per plant; g plant^–1^) was recorded, and the total number of seeds per plant was counted. Seeds per pod (SPP) was then calculated by dividing the total number of seeds by the total PN per plant. Individual seed weight (SW; g seed^–1^) was calculated by dividing the SY by the total number of seeds per plant. Harvest index (HI), the fraction of SDM allocated to the seed, was calculated as the SY divided by the SDM per plant. After harvesting the aboveground plant parts, the soil and the intact root systems within each rooting column were carefully removed by pulling out the translucent plastic liner after laying the tube down on its side. The rooting profile was then divided into five equal sections (0–20, 20–40, 40–60, 60–80, and 80–100 cm soil depths) by cutting from top to bottom with a large kitchen knife. Each root section was separately washed and placed into a labeled paper bag, so that root DM distribution could be determined by depth. All the root samples were oven-dried in a forced air drier at 80°C until a constant weight was attained (typically 4 days) and then final root DM (RDM) of each sample was recorded. Root-to-shoot DM ratio (R:S) was calculated as the ratio of RDM to SDM. Whole-plant DM-based water use efficiency (WUE; g L^–1^) was calculated by dividing whole-plant DM (TDM; RDM + SDM) by total WU from planting to full maturity.

### Statistical Analyses

All statistical analyses were performed using the PROC GLIMMIX procedure of SAS Version 9.4 (SAS Institute Inc., Cary, NC, United States). A Type 1 error rate of 0.05 was used for all statistical tests. Since the dependent variables SY, PN, SPP, SW, TDM, SDM, RDM, R:S, HI, WU, WUE, and VSWC were quantitative and continuous, a linear mixed model (LMM) was fitted. The variances of SY, PN, SPP, SW, TDM, SDM, RDM, R:S, HI, WU, and WUE were partitioned into the fixed effects of fertilizer placement and watering treatments, and their interactions (fertilizer × watering treatments), and the random effects of blocks. The following statistical model was used:

$$Y_{i\,j\,k}=\mu +f_{i}+w_{j}+f_{i}\,w_{j}+B_{k}+\varepsilon_{i\,j\,k}$$

where *Y*_*ijk*_ denotes the value of the measured trait for the *i*th fertilizer placement treatment (top loading or full loading) of the *j*th watering treatment (control, mild drought stress or drought stress) in the *k*th block, *μ* is the grand mean, *f*_*i*_ is the effect of the *i*th fertilizer placement treatment (the first factor), *w*_*j*_ is the effect of the *j*th watering treatment (i.e., the second factor), *f_*i*_w_*j*_* is the interaction effect between the *i*th fertilizer placement and the *j*th watering treatment, *B*_*k*_ is the effect of the *k*th block (treated as a random effect), and ε*_*ijk*_* is the residual.

The repeated measures analysis of RDM and VSWC by depth was partitioned into the fixed effects of fertilizer placement treatments, watering treatments, and depth, and their interactions (fertilizer × water, fertilizer × depth, water × depth, and fertilizer × water × depth), and the random effects of blocks. The random interaction term *subject × depth* was included in the model where the *subjects* (tubes) were assumed independent (identity covariance structure) and for *depth* three possible types of covariance structures [*compound symmetric*, CS; *autoregressive order 1*, AR(1); and heterogeneous autoregressive order 1, ARH(1)] were compared. In each case, the most appropriate model was selected based on AICC and BIC. *F*-tests and log-likelihood ratio tests were used to determine the significance of fixed and random effects, respectively. Since the RDM and VSWC measurements were equally spaced along the height of the tubes, the *Kenward-Roger* adjustment for bias correction for the denominator degrees of freedom was applied (Kenward and Roger, 1997). Least-square means were compared pairwise using Tukey’s test.

The assumptions for the LMM, in particular, random and normally distributed experimental errors and constant (homogeneous) error variance were tested (1) by plotting the studentized residuals against factor levels and predicted values, (2) by generating a Q-Q plot and scatterplots of the residuals vs. fitted values, and (3) by performing a formal test of normality using a Shapiro-Wilk. Putative outliers, if any, were detected if the values of the studentized residuals were not within the range of -3.4 to 3.4 (Bowley, 2015). The relationships between whole-plant WU and whole-plant DM, or SY were investigated via correlation and regression analyses using the PROC CORR and PROC REG procedures in SAS Version 9.4 (SAS Institute Inc., Cary, NC, United States).

## Results

### Effects of Fertilizer Placement and Drought Stress on Soybean Yield and Yield Components

**FIGURE 2:**
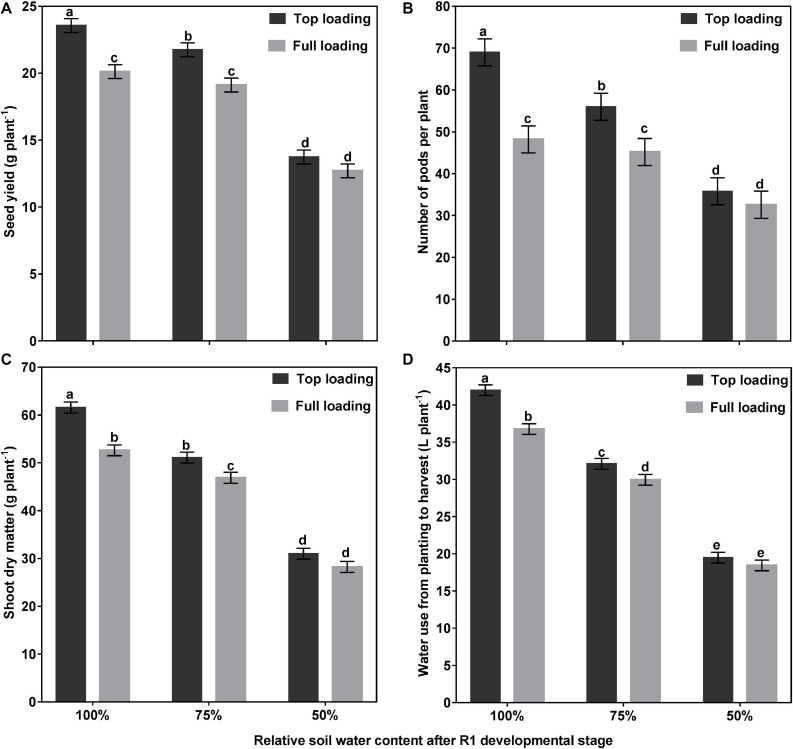
Interactive effects of fertilizer placement and drought stress treatments on seed yield per plant **(A)**, pods per plant **(B)**, shoot dry matter per plant **(C)**, and water use per plant **(D)** for soybean grown in a greenhouse in 1-m rooting columns. Fertilizer placement treatments are top loading or full loading, watered daily to either 100% soil water holding capacity (SWHC; control), 75% SWHC (mild drought stress), or 50% SWHC (drought stress). Data represent the fertilizer placement by drought stress treatment interaction least square mean values ± 1 S.E. Five replicates were used. Within a measured trait (panel), least-square means labeled with the same letter are not significantly different (*p* ≥ 0.05) according to a Tukey’s test.

Table 1 shows the main effects of fertilizer placement, watering treatment, and fertilizer by water interaction on soybean yield and yield components. Of the two fertilizer placement treatments tested, higher SY was obtained when the fertilizer was placed in the top 30 cm of the 1-m rooting columns. Averaged across the three watering treatments, the top loading (TL) fertilizer placement significantly increased SY by 14% (*p* < 0.0001) and PN by 24% (*p* < 0.001), as compared to the full loading (FL) fertilizer placement treatment. The fertilizer placement, however, did not affect SPP or SW (Table 1). Averaged across the two fertilizer placement treatments, the drought stress (50% SWHC) treatment significantly reduced SY by 40% (*p* < 0.0001), PN by 42% (*p* < 0.0001), and SPP by 3% (*p* < 0.05) as compared to the control (100% SWHC) watering treatment, but it did not affect SW. The mild drought stress (75% SWHC) treatment was intermittent between the other two watering treatments for SY and PN (Table 1). Moreover, there was a significant fertilizer placement by watering treatment interaction effect for SY (*p* < 0.01) and PN (*p* < 0.05) (Table 1 and Figure 2). When the fertilizer placement was FL, the 100 and 75% watering treatments did not differ for SY and PN. However, when the fertilizer placement was TL, the SY and PN were significantly higher under the 100% watering treatment than under the 75% watering treatment. There was no significant fertilizer effect on SY and PN under the 50% watering treatment (Figure 2).

**TABLE 1:** Effects of fertilizer placement, watering treatment, and their interaction on yield and yield components of soybean grown in a greenhouse in 1-m rooting columns under two fertilizer placement treatments (top loading or full loading) and three watering treatments [control (100% soil water holding capacity; SWHC), mild drought stress (75% SWHC), and drought stress (50% SWHC)] in 2016.

	**Seed yield**	**No. pods**	**No. seeds**	**Seed weight**
	**(g plant**^–^**^1^)**	**per plant**	**per pod**	**(g seed**^–^**^1^)**
**Fertilizer (F)**				
Top loading	19.7 a**^†^**	52.9 a	2.27 a	0.168 a
Full loading	17.3 b	42.7 b	2.37 a	0.172 a
S.E.	0.38	1.93	0.067	0.0045
*p* Fertilizer**^‡^**	**<0.0001**	**0.0008**	0.0860	0.5600
**Water (W)**				
100%	21.8 a**^†^**	58.6 a	2.33 ab	0.166 a
75%	20.4 b	50.6 b	2.38 a	0.170 a
50%	13.2 c	34.2 c	2.26 b	0.172 a
S.E.	0.42	2.33	0.04	0.006
*p* Water**^‡^**	**<0.0001**	**<0.0001**	**0.0239**	0.7192
*p* F **×** W**^‡^**	**0.0019**	**0.0174**	0.4865	0.3398
C.V. (%)	5.3	14.9	4.0	11.1

**^†^Within a factor (fertilizer or water) and column, least-square means followed by the same letter are not significantly different (p ≥ 0.05) according to a Tukey’s test.****^‡^****Significant fertilizer, water and fertilizer by water interaction effects (p < 0.05) are indicated in bold.**

### Effects of Fertilizer Placement and Drought Stress on Plant Growth Traits (DM, HI, WU, WUE, and Cumulative WU)

**FIGURE 3:**
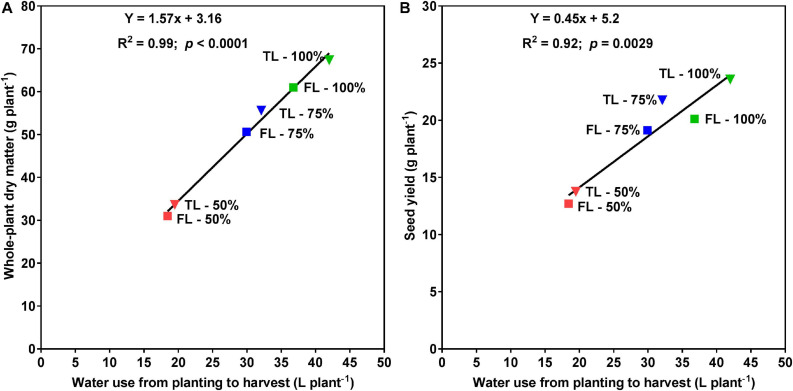
Relationships between whole-plant water use and whole-plant dry matter (**A**; left), or seed yield (**B**; right) for soybean grown in 1-m rooting columns in a greenhouse. Fertilizer placement treatments are top loading (TL; triangular symbols) or full loading (FL; square symbols), watered daily to either 100% soil water holding capacity (SWHC; control; green), 75% SWHC (mild drought stress; blue), or 50% SWHC (drought stress; red). The line is the best fit regression, not forced through the origin. Data represent the fertilizer placement by drought stress treatment interaction least square mean values. Five replicates were used.

**FIGURE 4:**
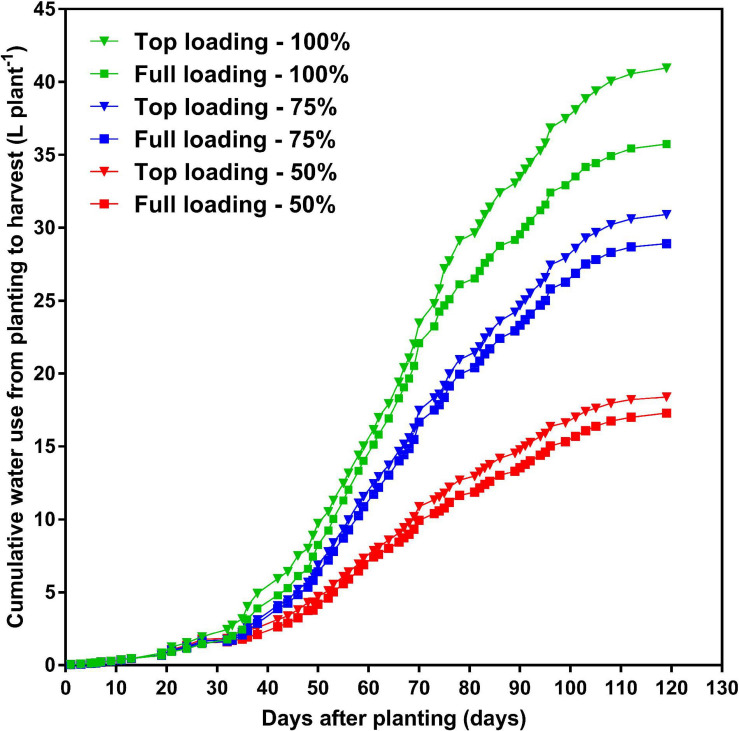
Effects of fertilizer placement and drought stress treatments on daily average cumulative water use from planting to harvest for soybean grown in a greenhouse in 1-m rooting columns. Fertilizer placement treatments are top loading (triangular symbols) or full loading (square symbols), watered daily to either 100% soil water holding capacity (SWHC; control; green), 75% SWHC (mild drought stress; blue), or 50% SWHC (drought stress; red). Data represent the fertilizer placement by drought stress treatment interaction least-square mean values. Five replicates were used.

At the R1 developmental stage, the TL fertilizer placement significantly (*p* < 0.0001) increased SDM by 60%, RDM by 40%, TDM by 55%, leaf area by 63%, and WU by 36% (data not shown). At full maturity, averaged across the three watering treatments, the TL fertilizer placement significantly (*p* < 0.0001) increased final SDM by 12%, TDM by 12%, and WU by 10%, as compared to the FL fertilizer placement; however, the fertilizer placement did not affect final RDM, R:S, HI, or WUE (Table 2). The drought stress treatment had a significant effect on every plant growth parameter measured. Averaged across the two fertilizer placement treatments, the 50% watering treatment significantly reduced SDM by 48% (*p* < 0.0001), RDM by 53% (*p* < 0.0001), TDM by 49% (*p* < 0.0001), and WU by 52% (*p* < 0.0001), as compared to the 100% watering treatment. The 75% watering treatment was intermittent between the other two watering treatments for these parameters. However, the 50% watering treatment significantly increased R:S by 23% (*p* < 0.01), HI by 17% (*p* < 0.0001), and WUE by 7% (*p* < 0.0001), as compared to the 100% watering treatment (Table 2). Moreover, there was a significant fertilizer placement by watering treatment interaction effect for SDM (*p* < 0.05) and WU (*p* < 0.01) (Table 2 and Figure 2). Under the 100 and 75% watering treatments, there was a significant fertilizer effect on SDM and WU. When the fertilizer placement was TL, the SDM and WU were significantly higher under the 100% watering treatment than under the 75% watering treatment. However, there was no significant fertilizer effect on SDM and WU under the 50% watering treatment (Figure 2). It should also be noted that the roots were extensively nodulated in both fertilizer placement treatments and the three watering treatments, although the seeds were not inoculated with rhizobia to promote nodulation. Whole-plant water use strongly predicted whole-plant dry matter accumulation (*R*^2^ = 0.99; *p* < 0.0001) and seed yield (*R*^2^ = 0.92; *p* = 0.0029) (Figure 3). The TL treatment had higher TDM accumulation and SY while the FL treatment had lower TDM accumulation and SY under the 100 and 50% watering treatments. The 75% watering treatment had intermittent TDM accumulation and SY under both fertilizer placement treatments. Figure 4 also shows the effects of fertilizer placement and drought stress treatments on apparent daily cumulative WU from planting to harvest as a function of days after planting. After the R1 developmental stage, the TL treatment had a higher daily cumulative WU than the FL treatment under all watering treatments (Figure 4). Before the R1 developmental stage, the TL and FL did not differ much for their daily cumulative WU.

**TABLE 2:** A generalized linear mixed model analysis of the effects of fertilizer placement, watering treatment, and their interaction on final shoot dry matter (DM), root DM, total DM, root-to-shoot DM ratio, harvest index, water use from planting to harvest, and whole-plant DM based water use efficiency of soybean grown in a greenhouse in 1-m rooting columns under two fertilizer placement treatments (top loading or full loading) and three watering treatments [control (100% soil water holding capacity; SWHC), mild drought stress (75% SWHC), and drought stress (50% SWHC)] in 2016.

	**Shoot DM**	**Root DM**	**Total DM**	**Root:shoot DM ratio**	**Harvest index**	**Water use**	**Water use efficiency**
	**(g plant**^–^**^1^)**	**(g plant**^–^**^1^)**	**(g plant**^–^**^1^)**	**(g g**^–^**^1^)**	**(g g**^–^**^1^)**	**(L plant**^–^**^1^)**	**(g L**^–^**^1^)**
**Fertilizer (F)**							
Top loading	47.9 a**^†^**	4.3 a	52.2 a	0.089 a	0.42 a	31.2 a	1.69 a
Full loading	42.6 b	4.1 a	46.7 b	0.096 a	0.41 a	28.4 b	1.65 a
S.E.	0.70	0.83	0.78	0.003	0.006	0.53	0.017
*p* Fertilizer**^‡^**	**<0.0001**	0.3598	**<0.0001**	0.1103	0.7736	**<0.0001**	0.1296
**Water (W)**							
100%	57.1 a**^†^**	5.8 a	62.9 a	0.082 b	0.38 c	39.4 a	1.59 b
75%	49.0 b	4.1 b	53.1 b	0.093 a	0.42 b	31.0 b	1.70 a
50%	29.6 c	2.7 c	32.3 c	0.101 a	0.45 a	19.0 c	1.71 a
S.E.	0.83	0.21	0.94	0.004	0.005	0.58	0.020
*p* Water**^‡^**	**<0.0001**	**<0.0001**	**<0.0001**	**0.0052**	**<0.0001**	**<0.0001**	**0.0005**
*p* F **×** W**^‡^**	**0.0266**	0.2564	0.0510	0.2188	0.4904	**0.0078**	0.9373
C.V. (%)	5.4	15.1	5.7	12.6	3.5	4.6	3.7

**A 2****×****3 factorial design with five replicates was used.****^†^****Within a factor (fertilizer or water) and column, least-square means followed by the same letter are not significantly different (p ≥ 0.05) according to a Tukey’s test.****^‡^****Significant fertilizer, water and fertilizer by water interaction effects (p < 0.05) are indicated in bold.**

### Effects of Fertilizer Placement on VSWC and Rooting Profiles by Depth Under Control Conditions (Pre-R1)

**FIGURE 5:**
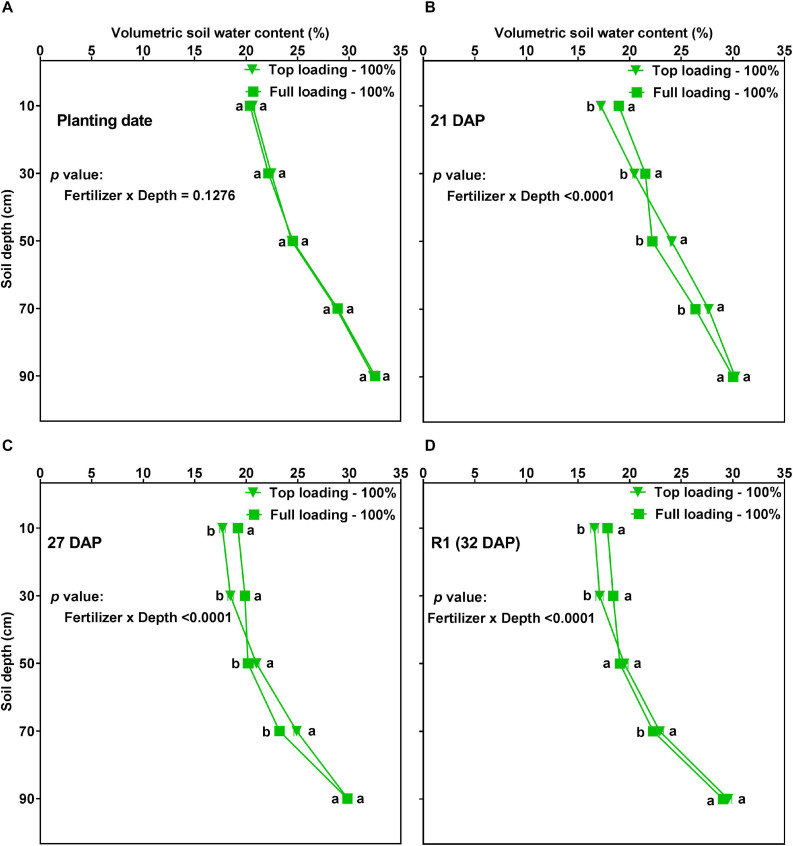
Volumetric soil water content (VSWC; %) by depth for soybean grown in a greenhouse in 1-m rooting columns under two fertilizer placement treatments [top loading (triangular symbols) or full loading (square symbols)] and watered daily to 100% soil water holding capacity. The VSWC measurements were taken 24 h after the previous watering. The measurements were made at planting date (**A**; top left), 21 days after planting (DAP) (**B**; top right), 27 DAP (**C**; bottom left), and 32 DAP (**D**; bottom right). Data represent the least square mean values of 15 plants ± 1 S.E. in each fertilizer placement treatment. Within a soil depth, values labeled with the same letter are not significantly different (*p* ≥ 0.05) according to a Tukey’s test. If not seen, the standard error is smaller than the symbol.

On several dates before the R1 developmental stage (i.e., before the initiation of the drought stress treatments), there was a significant (*p* < 0.0001) fertilizer placement by depth interaction effect for VSWC. The effect of depth on VSWC differed between the fertilizer placement treatments at 21, 27, and 32 DAP, but not on the planting date (Figure 5). That is, the TL and FL did not significantly differ in their VSWC by depth before the plants had accessed the water and fertilizer in the soil (Figure 5A). However, at 21, 27, and 32 DAP, the plants under the TL treatment showed more water depletion in the top part of the soil profile while the plants under the FL showed more water depletion from the mid and lower parts of the soil profile (Figures 5B–D). The treatments had similar VSWC at the very bottom part of the profile (90 cm depth) at these developmental stages. Thus, in the pre-R1 period, fertilizer placement affected the VSWC profile by depth.

**FIGURE 6:**
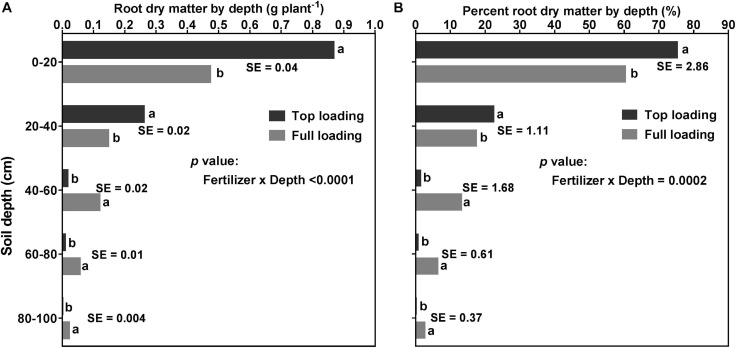
Effect of fertilizer placement treatment and soil depth on root dry matter distribution (**A**; left) and percent root dry matter distribution (**B**; right) for 32-day-old soybean plants grown in a greenhouse in 1-m rooting columns. Fertilizer placement treatments are top loading or full loading, watered daily to 100% soil water holding capacity. Data are from a destructive harvest made at 32 days after planting (R1 developmental stage), prior to the initiation of the drought stress treatments. Each data point at each soil profile depth in each fertilizer placement treatment represents the least square mean value of 15 plants ± 1 S.E. Within a soil depth, values followed by the same letter are not significantly different (*p* ≥ 0.05) according to a Tukey’s test. There was a significant (*p* < 0.05) two-way interaction (fertilizer × depth) effect for root dry matter and percent root dry matter distribution by depth.

Figure 6 shows that there was a significant (*p* < 0.0001) fertilizer placement by depth interaction effect for RDM and percent RDM distribution, at the R1 developmental stage. Fertilizer placement treatments ranked differently at different depths of the profile. Compared to the FL, plants in the TL treatment had significantly higher RDM and percent RDM in the top 0–40 cm depth but lower RDM and percent RDM starting from the middle to the bottom part of the soil profile (i.e., 40–100 cm depth) (Figure 6).

### Effects of Fertilizer Placement and Drought Stress on VSWC and Rooting Profiles by Depth (Post-R1)

**FIGURE 7:**
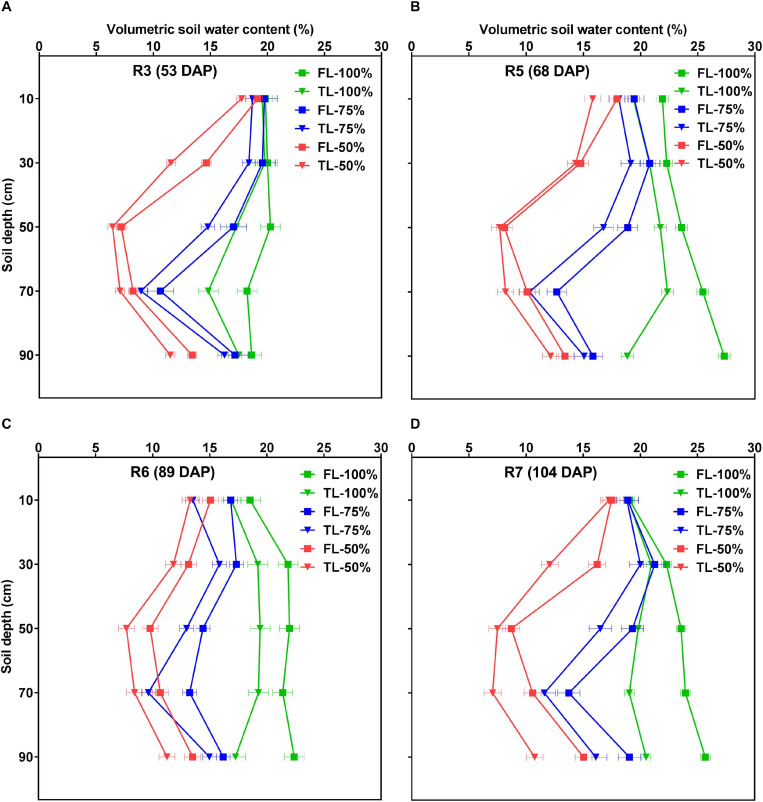
Effects of fertilizer placement, watering treatment, and soil depth on volumetric soil water content (VSWC; %) for soybean grown in a greenhouse in 1-m rooting columns. Fertilizer placement treatments are top loading (TL) or full loading (FL), watered daily to either 100% soil water holding capacity (SWHC; control), 75% SWHC (mild drought stress), or 50% SWHC (drought stress). The VSWC measurements were taken 24 h after the previous watering. The measurements were made at the R3 (**A**; top left), R5 (**B**; top right), R6 (**C**; bottom left), and R7 (**D**; bottom right) developmental stages. Drought stress treatments were imposed at the R1 developmental stage. Data represent the fertilizer × water × depth least-square mean values ± 1 S.E. Five replicates were used. If not seen, the standard error is smaller than the symbol. There were no significant fertilizer × water, fertilizer × depth, or fertilizer × water × depth interaction effects for VSWC. However, there were significant fertilizer, water, depth, and water × depth effects for VSWC.

Figure 7 and Supplementary Figure 1 show the effects of fertilizer placement, watering treatment, and soil depth on VSWC across different developmental stages. At all developmental stages, there were statistically significant (*p* < 0.0001) effects of fertilizer placement, watering treatment, depth, and water × depth for VSWC but there were no significant two-way (fertilizer × water, fertilizer × depth), or three-way (fertilizer × water × depth) interaction effects. In other words, the TL fertilizer placement resulted in significantly more water use, resulting in lower VSWC than the FL fertilizer placement treatment, and this difference was similar across soil depth × watering treatment combinations within each developmental stage. The overall pattern of soil water depletion was consistent for the two fertilizer placement treatments for most of the profile depths and developmental stages within each watering treatment (Figure 7). Averaged across the three watering treatments and five profile depths, the TL fertilizer placement treatment had a significantly (*p* < 0.0001) lower VSWC in the rooting columns than the FL at all developmental stages, after the initiation of the drought stress treatment (Supplementary Table 1).

Once the watering treatments were imposed (after the R1 developmental stage), there were also clear differences in VSWC profiles among the three watering treatments throughout the remainder of the experiment (Supplementary Figure 1). That is, averaged across the two fertilizer placement treatments, there were significant (*p* < 0.0001) differences for VSWC by depth among the three watering treatments. The 100% watering treatment had a significantly higher VSWC by depth (a wetter profile) followed by the 75% watering treatment. As expected, the 50% watering treatment resulted in the lowest VSWC (the driest profile) of the three watering treatments, and this effect increased as plants grew larger and daily water use increased. Compared to the other two watering treatments, the 100% watering treatment resulted in a quite uniform VSWC over most of the 1-m depth, and with the smallest change in VSWC between the bottom and top, providing more available water for plant growth (Supplementary Figure 1). For the same difference in gravitational potential (0.01 MPa from the top of the tube to the bottom), there was a larger change in VSWC and therefore probably less plant available water in the 50% watering treatment, while the 75% watering treatment fell in between the other two watering treatments (Supplementary Figure 1).

**FIGURE 8:**
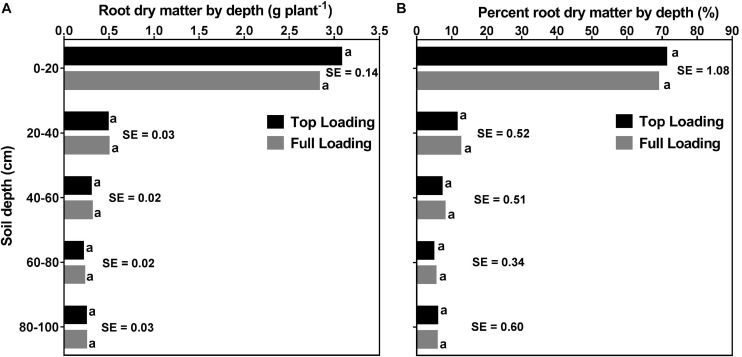
Effects of fertilizer placement and soil depth on root dry matter distribution (**A**; left) and percent root dry matter distribution (**B**; right) for soybean grown in a greenhouse in 1-m rooting columns. Fertilizer placement treatments are top loading or full loading. Data are from a harvest measurement made at physiological maturity (120 days after planting). Each data point at each soil profile depth represents fertilizer placement treatment (averaged across three watering treatments) least square mean values ± 1 S.E. Within a soil depth, fertilizer least-square means followed by the same letter are not significantly different (*p* ≥ 0.05) according to a Tukey’s test. Five replicates were used.

**FIGURE 9:**
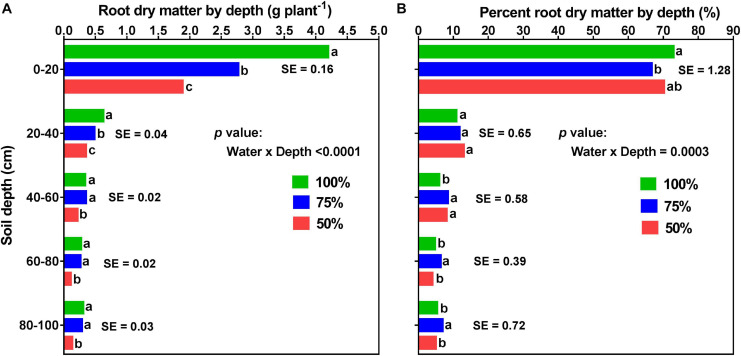
Effects of watering treatment and soil depth on root dry matter (**A**; left) and percent root dry matter distribution (**B**; right) for soybean grown in a greenhouse in 1-m rooting columns. Drought stress treatments are watering daily to either 100% soil water holding capacity (SWHC; control), 75% SWHC (mild drought stress), or 50% SWHC (drought stress). Data are from a harvest measurement made at physiological maturity (120 days after planting). Drought stress was imposed at the R1 developmental stage. Each data point at each soil profile depth represents drought stress treatment (averaged across the two fertilizer placement treatments; top loading or full loading) least square mean values ± 1 S.E. Within a soil depth, drought stress treatment least-square means followed by the same letter are not significantly different (*p* ≥ 0.05) according to a Tukey’s test. Five replicates were used.

Figures 8, 9 display the effects of fertilizer placement, watering treatment, and soil depth on final RDM and percent RDM distribution by depth. There were significant (*p* < 0.0001) effects of watering treatment, depth, and water × depth but there were no significant effects of fertilizer placement, fertilizer × water, fertilizer × depth, or fertilizer × water × depth for RDM distribution. Additionally, there were significant (*p* < 0.0001) effects of depth and water × depth but there were no significant effects of fertilizer placement, watering treatment, fertilizer × water, fertilizer × depth, or fertilizer × water × depth for percent RDM distribution. At full maturity, there was no significant effect of fertilizer placement on RDM and percent RDM distribution by depth (Table 2 and Figure 8), in contrast to what was observed pre-R1 (Figure 6).

Averaged across the two fertilizer placements and three watering treatments, there were significant differences between the five profile depths for both root and percent root DM. That is, a significantly higher RDM was allocated to the top part of the profile (0–20 cm; 2.95 ± 0.089 g) followed by 20–40 cm (0.49 ± 0.022 g) and 40–60 cm (0.30 ± 0.013 g) depths. The two bottom parts of the profile (60–80 cm and 80–100 cm depths) had lower but statistically similar RDM, 60–80 cm (0.21 ± 0.012 g) and 80–100 cm (0.24 ± 0.019 g) (Supplementary Table 2). Likewise, a significantly higher percent RDM was allocated to the top part of the profile (0–20 cm; ∼70%) followed by the 20–40 cm (∼12%), and the 40–60 cm (∼8%) profile depths. The two bottom profile depths, 60–80 cm, and 80–100 cm did not differ from each other, each contributing about 5% of the RDM distribution (Supplementary Table 3).

Averaged across the two fertilizer placements, there were significant (*p* < 0.0001) differences in RDM among the three watering treatments at different profile depths. Drought stress significantly altered RDM and percent RDM distribution by depth (Figure 9). For all the three watering treatments, more than 65% of the total RDM was distributed in the upper 20 cm of the soil profile; 73% for the control, 70% for the drought stress, and 67% for the mild drought stress treatments. However, the two drought stress treatments, especially the mild drought stress treatment, had a greater proportion of the total RDM located in the deeper soil layers (i.e., 60–80 and 80–100 cm); yet, there was no difference between these two watering treatments at the 0–20, 20–40, and 40–60 cm depths of the profile (Figure 9B).

## Discussion

A primary objective of this work was to develop and test a drought stress simulation protocol in 1-m rooting columns, that would create consistent, repeatable reductions in soybean water use, growth, and yield. This was achieved; there were clear differences among the three watering treatments for most plant growth and yield parameters such as SY, PN, SDM, RDM, TDM, WU, and HI. Additionally, it was important that the yield reduction be associated with the specific yield component changes that typically characterize drought-induced yield losses in field-grown soybean. We found that SW was not responsive to either watering treatment and SPP also did not vary substantially between the control and drought stress treatments. Instead, PN was the most important yield component driving SY response to drought stress, as is typically observed in the field (e.g., Shaw and Laing, 1966; Momen et al., 1979; He et al., 2017).

Also importantly, the drought stress protocol from this experiment did not produce symptoms of severe stress, such as leaf wilting, that are usually not observed under Ontario field conditions. Taken together, these results suggest that the drought stress protocol in this work was a realistic simulation of how soil water deficits in the field reduce soybean growth and yield.

The drought stress (50% maximum SWHC) treatment produced effects on DM and SY that are of an appropriate magnitude for future genotype comparisons. Moreover, the overall coefficients of variation (C.V.) were low, at 5.3 and 5.7% for SY and TDM, respectively, suggesting a very consistent yield reduction and a good repeatability of the treatments. This would offer very good statistical power for future phenotyping studies to detect genotype differences for traits related to drought stress tolerance.

Another objective for this system was to achieve a below-ground environment that allowed for root biomass distribution by depth similar to what occurs in the field. The rooting profiles observed in this study were quite similar to those that have been observed in the field and in other controlled environment studies, where the majority of the roots (>50%) are located in the upper (e.g., 0–20 cm) soil layer under both control and drought stress conditions, for example, in soybean (Dwyer et al., 1988; Kirkham et al., 1998; Benjamin and Nielsen, 2006; Ordonez et al., 2018; Gebre, 2020; Gebre and Earl, 2020) and maize [*Zea mays* L. (Dwyer et al., 1988; Kirkham et al., 1998; Ordonez et al., 2018; Zhang et al., 2018)].

As expected, both drought stress treatments significantly increased R:S and WUE, with the effects being stronger under the severe drought stress treatment. These effects are consistent with a growth strategy that prioritizes both acquisition and efficient utilization of water under drought stress. Similar observations of increased R:S under drought stress have been reported previously (e.g., Boutraa et al., 2010; Gebretsadik, 2011; Ehdaie et al., 2012; Wang et al., 2014). Earl (2002) in soybean also found higher plant WUE in a drought stress treatment, while Hufstetler et al. (2007) reported no significant difference in WUE of soybean between drought stress and control watering treatments. An unexpected result of the present work was the increased HI under drought stress. Contrary to this, reduced HI under drought stress has been reported in maize (Earl and Davis, 2003) and soybean (Oya et al., 2004; Giordani et al., 2019).

We predicted that the fertilizer placement would change the RDM and VSWC by depth. That is, placing the fertilizer throughout the soil profile would result in more roots at depth which would, in turn, enhance the plants’ ability to extract soil water from the lower depths and therefore be more drought tolerant. At R1, we observed this predicted effect of fertilizer placement on root biomass distribution and soil water extraction. Similar to our results, Hansel et al. (2017) also reported an increased root growth in soybean as evidenced by a significantly higher root length density (RLD) in deeper soil layers when the fertilizer was placed deeper in the soil profile. On the other hand, they also reported a relatively shallower root growth with a greater proportion (>60%) of roots distributed in the upper soil layer when the fertilizer was placed in the upper soil profile, similar to what was observed in our TL fertilizer placement treatment. However, contrary to our results at full maturity, Hansel et al. (2017) found that deeper fertilizer placement methods improved soybean drought tolerance by promoting deeper plant root systems, thereby enhancing water acquisition. Singh et al. (2005) also found that deeper fertilizer placement increased shoot dry matter and grain yield of spring wheat (*Triticum aestivum* L.), possibly by preventing the condition where nutrients in upper soil layers became unavailable to plants when those layers dried (i.e., drought-induced nutrient deficiency). However, Singh et al. (2005) did not measure RDM distribution or soil water extraction by depth, so it is not known if their fertilizer placement treatments affected root growth or access to soil water.

In our study, the effects of fertilizer placement on soil water extraction at depth did not persist until maturity, and we did not find an advantage of deep fertilizer placement with respect to enhancing drought tolerance. However, our protocol was not ideal for generating a soil water “reward” in response to deep rooting; deeper rooting did not bring a permanent advantage in terms of soil water access because we replaced the water used by each plant every 24 h regardless. Also, the bottom part of the soil profile became relatively dry in all cases when plants were large, because the water added to the tops of the tubes with daily watering, enough to achieve the target weight, was partially transpired before it could percolate to the bottom part of the profile. Instead, our system was well-designed to assess where the roots were and where they were actively extracting water in the soil profile. The fact that we were able to detect as statistically significant small fertilizer placement effects on root activity in terms of soil water extraction suggests that this phenotyping system could be useful for investigating how genotypes differ for small differences in root activity by depth. In other words, if there were genotype differences in soil water extraction by depth at the R1 developmental stage, we should be able to detect those effects in this system, even for a small difference (e.g., 5%); also, the measurement is non-destructive, and so can be repeated on the same experimental units over time.

The FL treatment appeared to create a nutrient deficiency relative to the TL treatment, as evidenced by significantly reduced SY, PN and TDM. The advantage of the TL treatment may have arisen from improved plant nutrient uptake when the fertilizer was concentrated in the same layer of the profile where the majority (over 65%) of the roots were distributed. However, the absolute size of the fertilizer placement effect on SY, PN, SDM, and WU was strongly dependent on the watering treatment; when water was not limiting, the advantage of TL over FL was larger than it was under drought stress. It appears that in the FL treatment, nutrient deficiency prevented the plants from making full use of the water-replete conditions; that is, soil water was less limiting to growth in the presence of a nutrient deficiency. As with the main effect of the water treatment, PN also clearly drove the fertilizer × water interaction for SY; there was no fertilizer x water interaction effect for SPP or SW, while 91% of the variation in SY could be explained by PN (analysis not shown).

Although Vadez et al. (2008) and Ratnakumar et al. (2009) have described a similar phenotyping system in terms of the rooting environment (they used 1 m long 20 cm diameter PVC tubes), there are some important differences between those past studies and the present work. First, their watering protocols followed the common approach of providing the same amount of water to every plant within a treatment, regardless of the plant size or current transpiration rate, whereas we tried to equalize actual VSWC between experimental units daily so that all plants within a treatment experienced a similar soil water deficit. Additionally, in the present work we monitored the soil water profile in the rooting columns and documented how it changed across different developmental stages. This allowed us to detect differences in soil water extraction by depth, especially as the roots progressively explored the lower part of the profile. This method should be useful for future phenotyping studies investigating root activity.

In this experiment, whole-plant water use strongly correlated with whole-plant DM accumulation (*R*^2^ = 0.99) across all fertilizer placement and watering treatments. This suggests that WU measurement can serve as a reasonable proxy for real-time whole-plant DM accumulation (growth) without requiring a destructive harvest. The linear association between SY and WU was slightly weaker (*R*^2^ = 0.92) as would be expected if WU primarily predicts TDM, since the association between WU and SY is weakened by any variation in HI.

A limitation of the current study is that there was only a single genotype included, and so it is not possible to know if the responses are genotype-specific. However, subsequently, we have employed this methodology in a phenotyping study to compare fifteen Ontario-adapted commercial elite varieties for their drought tolerance. Although responses of the genotypes to the soil water deficit were qualitatively similar across the genotypes, we were able to detect quantitative genotypic differences in drought tolerance in terms of their growth, water use, seed yield and yield component responses to the soil water deficit using this phenotyping system (Gebre, 2020).

## Conclusion

In this study we developed, evaluated and characterized a unique drought stress simulation method for controlled environment phenotyping of soybean varieties, that has a more realistic belowground environment (1-m rooting columns) than can be achieved in small pot experiments. It produced drought responses that are large enough to easily measure, and the type of physiological changes in yield and yield components, DM, WU, WUE, and rooting profiles that are expected for soybean encountering drought stress in the field. This system can also detect non-destructively small in-season differences between treatments (e.g., fertilizer placements in this case) in soil water extraction by depth. This system should be ideal for comparing genotypes for their root activity; it could be useful to select for root function and yield formation traits that could decrease soybean yield losses under drought stress conditions.

Our results suggest that the 50% maximum SWHC drought stress protocol was the best treatment for controlled-environment phenotyping studies. Drought stress treatments imposed in this culture system affected yield components in a manner similar to what is observed in the field, with PN being the yield component most strongly affected. The C.V. for SY was low, at 5.3%, suggesting a very consistent yield reduction and a good repeatability of the treatments, and potentially offering good statistical power for investigating genotype differences in abiotic stress responses. In addition to exploring the effects of drought stress on shoot traits, this novel phenotyping system provides an opportunity to investigate final RDM distribution in the profile, as well as soil water extraction from different profile strata at any developmental stage.

## Data Availability Statement

All datasets generated for this study are included in the article/Supplementary Material, further inquiries can be directed to the corresponding author/s.

## Author Contributions

MG performed the experiment, data collection, statistical data analysis and presentation, and drafted the manuscript. MG and HE conceived the project and experimental design and collaborated on the data interpretation and manuscript revision. Both authors contributed to the article and approved the submitted version.

## Conflict of Interest

The authors declare that the research was conducted in the absence of any commercial or financial relationships that could be construed as a potential conflict of interest.

## Footnotes

1 www.secan.com
