# Supplementary material for: Soil Water Deficit and Fertilizer Placement Effects on Root Biomass Distribution, Soil Water Extraction, Water Use, Yield, and Yield Components of Soybean [Glycine max (L.) Merr.] Grown in 1-m Rooting Columns
Source: Front Plant Sci. 2021 Mar 15;12:581127. doi: 10.3389/fpls.2021.581127 (PMC8005719; doi:10.3389/fpls.2021.581127)
Supplement: Supplementary file 1 [file Data_Sheet_1.docx]

Supplementary Material

# Supplementary Tables and Figures

## Supplementary Tables

**Supplementary Table 1:** A generalized linear mixed model repeated measures analysis of the effects of fertilizer placement, watering treatment, and their interaction on volumetric soil water content (VSWC; %) for soybean grown in a greenhouse in 1-m rooting columns under two fertilizer placement treatments (Top loading or Full loading) and three watering treatments [control (100% soil water holding capacity; SWHC), mild drought stress (75% SWHC), and drought stress (50% SWHC)] in 2016. The VSWC measurements were taken 24 h after the previous watering. The measurements were made at R3, R5, R6, and R7 developmental stages. Drought stress was imposed at the R1 developmental stage. Data represent the fertilizer placement treatment (averaged across the three drought stress treatments) and the drought stress treatment (averaged across the two fertilizer placement treatments) least square mean values ± 1 S.E. A 2 **×** 3 factorial design with five replicates was used.

|  | VSWC (%) at different developmental stages | | | |
| --- | --- | --- | --- | --- |
|  | R3 | R5 | R6 | R7 |
| Fertilizer (F) |  |  |  |  |
| Full loading | 16.2 a**^†^** | 18.2 a | 16.4 a | 18.3 a |
| Top loading | 14.7 b | 16.1 b | 14.1 b | 15.8 b |
| S.E. | 0.25 | 0.15 | 0.26 | 0.18 |
| *p* Fertilizer**^‡^** | **<0.0001** | **<0.0001** | **<0.0001** | **<0.0001** |
|  |  |  |  |  |
| Water (W) |  |  |  |  |
| 100% | 18.6 a**^†^** | 22.4 a | 19.8 a | 21.4 a |
| 75% | 16.1 b | 16.7 b | 14.5 b | 17.5 b |
| 50% | 11.7 c | 12.3 c | 11.5 c | 12.3 c |
| S.E. | 0.30 | 0.24 | 0.31 | 0.25 |
| *p* Water**^‡^** | **<0.0001** | **<0.0001** | **<0.0001** | **<0.0001** |
|  |  |  |  |  |
| *p* F **×** W**^‡^** | 0.9458 | 0.0547 | 0.5381 | 0.3294 |

**^†^**Within a factor (fertilizer or water) and column, least-square means followed by the same letter are not significantly different (*p* ≥ 0.05) according to a Tukey’s test. **^‡^**Significant fertilizer, water and fertilizer by water interaction effects (*p* < 0.05) are indicated in bold.

**Supplementary Table 2:** A generalized linear mixed model repeated measures analysis of the effects of soil depth on root dry matter (DM) for soybean grown in a greenhouse in 1-m rooting columns under two fertilizer placement treatments (top loading or full loading) and three watering treatments [control (100% soil water holding capacity; SWHC), mild drought stress (75% SWHC), and drought stress (50% SWHC)] in 2016. Data represent the depth (averaged across two fertilizer placement and three watering treatments) least square mean values ± 1 S.E. A 2 **×** 3 factorial design with five replicates was used.

| Soil depth (cm) | Root DM (g plant^-1^) | Standard error |
| --- | --- | --- |
| 0-20 | 2.95 a**^†^** | 0.089 |
| 20-40 | 0.49 b | 0.022 |
| 40-60 | 0.30 c | 0.013 |
| 60-80 | 0.21 d | 0.012 |
| 80-100 | 0.24 d | 0.019 |

**^†^**Within a column, least-square means followed by the same letter are not significantly different (*p* ≥ 0.05) according to a Tukey’s test.

**Supplementary Table 3:** A generalized linear mixed model repeated measures analysis of the effects of soil depth on percent root dry matter (DM) for soybean grown in a greenhouse in 1-m rooting columns under two fertilizer placement treatments (top loading or full loading) and three watering treatments [control (100% soil water holding capacity; SWHC), mild drought stress (75% SWHC), and drought stress (50% SWHC)] in 2016. Data represent the depth (averaged across two fertilizer placement and three watering treatments) least square mean values ± 1 S.E. A 2 **×** 3 factorial design with five replicates was used.

| Soil depth (cm) | Root DM (%) | Standard error |
| --- | --- | --- |
| 0-20 | 69.9 a**^†^** | 0.74 |
| 20-40 | 11.9 b | 0.41 |
| 40-60 | 7.5 c | 0.33 |
| 60-80 | 5.0 d | 0.24 |
| 80-100 | 5.7 d | 0.42 |

**^†^**Within a column, least-square means followed by the same letter are not significantly different (*p* ≥ 0.05) according to a Tukey’s test.

## Supplementary Figures

**
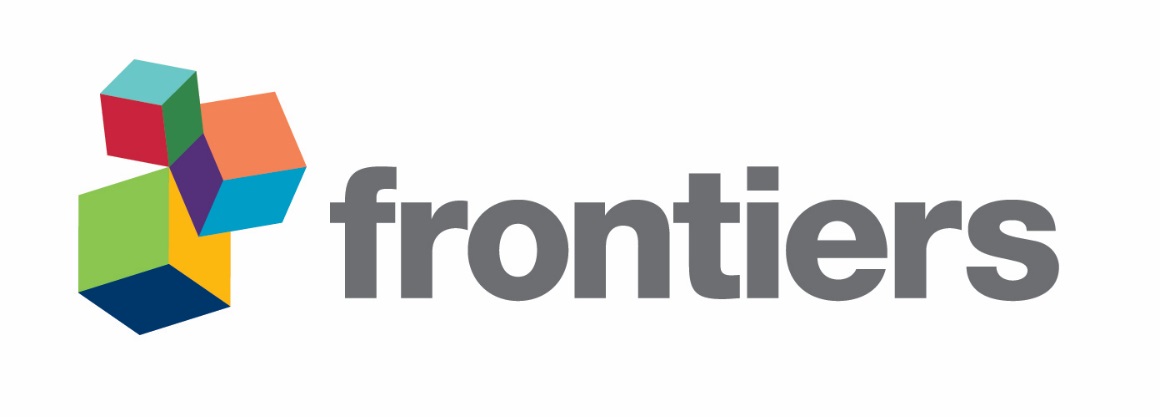
**

**Supplementary Figure 1:** Effects of watering treatment and soil depth on volumetric soil water content (VSWC; %) for soybean grown in a greenhouse in 1-m rooting columns in 2016. Drought stress treatments are watering daily to either 100% soil water holding capacity (SWHC; control), 75% SWHC (mild drought stress), or 50% SWHC (drought stress). The VSWC measurements were taken 24 h after the previous watering. The measurements were made at the R3 (panel A; top left), R5 (panel B, top right), R6 (panel C; bottom left), and R7 (panel D; bottom right) developmental stages. Drought stress was imposed at the R1 developmental stage. Data represent the water **×** depth (averaged across two fertilizer placement treatments; top loading or full loading) least square mean values ± 1 S.E. in each drought stress treatment. Five replicates were used. If not seen, the standard error is smaller than the symbol. There were no significant fertilizer **×** water, fertilizer **×** depth, or fertilizer **×** water **×** depth interaction effects for VSWC. However, there was a significant water **×** depth interaction effect for VSWC.
